# Retraction Note: The function of Cav-1 in MDA-MB-231 breast cancer cell migration and invasion induced by ectopic ATP5B

**DOI:** 10.1007/s12032-025-02623-6

**Published:** 2025-02-08

**Authors:** Xinjie Dong, Yilei Li, Wei Li, Wenzhe Kang, Rong Tang, Wenyi Wu, Ziyi Xing, Lijuan Zhou

**Affiliations:** 1 Department of Pathology, The First Hospital of Jilin University, Changchun, 130021 Jilin China; 2 The Key Laboratory of Pathobiology, Ministry of Education, The College of Basic Medical Sciences, Jilin University, Changchun, 130021 Jilin China; 3 Electron Microscopy Laboratory of Renal Pathology, The First Affiliated Hospital of Zhengzhou University, Zhengzhou, 450052 Henan China

**Retraction Note to: Medical Oncology (2021) 38:73** 10.1007/s12032-021-01519-5

The Editor in Chief has retracted this article on request from the authors because the Migration/B04 panel in Fig. 3A overlaps with the Invasion/Cav-1 shRNA + Chol panel in Fig. 7A which reports a different experiment. The results are therefore unreliable.

The Authors Xinjie Dong, Yilei Li, Wei Li, Wenzhe Kang, Rong Tang, Wenyi Wu and Lijuan Zhou have not responded to correspondence from the editor or publisher about this retraction. The Publisher was not able to contact Author Ziyi Xing.

